# The Clock’N Test as a Possible Measure of Emotions: Normative Data Collected on a Non-clinical Population

**DOI:** 10.3389/fnbeh.2016.00008

**Published:** 2016-02-05

**Authors:** Auriane Gros, Valeria Manera, Anaïs Daumas, Sophie Guillemin, Olivier Rouaud, Martine Lemesle Martin, Maurice Giroud, Yannick Béjot

**Affiliations:** ^1^Department of Neurology, University Hospital of Dijon and EA4184 of the University of BurgundyDijon, France; ^2^CoBTeK Team (Cognition Behavior Technology), Institut Claude Pompidou, University of Nice Sophia AntipolisNice, France; ^3^Dijon Stroke Registry, University Hospital and Medical School of Dijon, University of BurgundyDijon, France; ^4^Resource and Research Memory Center, Hospital of DijonDijon, France; ^5^Neurophysiology Department of DijonDijon, France

**Keywords:** emotional disorders, priming effect, skin conductance, time estimation, neuropsychological test

## Abstract

**Objective:** At present emotional experience and implicit emotion regulation (IER) abilities are mainly assessed though self-reports, which are subjected to several biases. The aim of the present studies was to validate the Clock’N test, a recently developed time estimation task employing emotional priming to assess implicitly emotional reactivity and IER.

**Methods:** In Study 1, the Clock’N test was administered to 150 healthy participants with different age, laterality and gender, in order to ascertain whether these factors affected the test results. In phase 1 participant were asked to judge the duration of seven sounds. In phase 2, before judging the duration of the same sounds, participants were presented with short arousing video-clip used as emotional priming stimuli. Time warp was calculated as the difference in time estimation between phase 2 and phase 1, and used to assess how emotions affected subjective time estimations. In study 2, a representative sample was selected to provide normative scores to be employed to assess emotional reactivity (Score 1) and IER (Score 2), and to calculate statistical cutoffs, based on the 10th and 90th score distribution percentiles.

**Results:** Converging with previous findings, the results of study 1 suggested that the Clock’N test can be employed to assess both emotional reactivity, as indexed by an initial time underestimation, and IER, as indexed by a progressive shift to time overestimation. No effects of gender, age and laterality were found.

**Conclusions:** These results suggest that the Clock’N test is adapted to assess emotional reactivity and IER. After collection of data on the test discriminant and convergent validity, this test may be employed to assess deficits in these abilities in different clinical populations.

## Introduction

Despite the disparate number of definitions (Kleinginna and Kleinginna, 1981), it is widely accepted that emotions are complex sets of reactions consisting of three components: (1) a behavioral/expressive component, indexed by emotional behaviors and emotional facial and bodily expressions; (2) a cognitive/experienced component, indexed by self-reported emotional feelings; and (3) a physiological/autonomic component, indexed by changes in the heart rate, electrodermal response (or skin conductance), body temperature and respiratory rate (Dimberg, 1987). These three emotion components are often measured separately, and employing different tests and tasks. For instance, the cognitive/experienced component is mainly assessed though self-report instruments, such as interviews or self-report questionnaires, which can evaluate either the person’s *emotional experience/reactivity* (self-reported perceived emotions) or her *emotion regulation (ER)* abilities (ability to up- or down-regulate the perceived emotions).

Separating these three components facilitates the study of emotions. However, the distinction is artificial, and the three elements are strongly interrelated and they influence each others in several bidirectional ways. For instance, experiencing emotions—such as fear—and displaying them through facial expressions activates consistently the autonomic system, as indexed, for instance, by an increase in skin conductance, with more intense emotional experience resulting in increased physiological activations (Christie and Friedman, 2004). Similarly, adopting the facial expression of an emotion can contribute to the feeling of the corresponding emotion (Buck, 1980), as well as to the activation of the corresponding physiological system (Levenson et al., 1990). Thus, designing tasks that can tap into several different components is an important research priority.

In the present article, we will present normative data for a recently developed instrument, the Clock’N test (Gros et al., 2015). Based on an emotional priming time estimation task, this test allows to assess *implicitly* the emotional experience and the ER linked to the cognitive/experiential component of emotions, while tapping into the physiological emotion component. In the next sections, we will first describe the main tasks currently employed to measure emotional experience and ER. Next, we will describe how time estimation works, and how it is influenced by emotions. Finally, we will briefly describe the Clock’N Test.

### Emotional Experience

Emotional experience is usually assessed through self-report rating scales in which the participant needs to assess to what degree he is feeling specific emotions. For instance, based on a categorical emotion approach (e.g., Ekman, 1992), the Differential Emotions Scale (DES; Ouss et al., 1990), the Brief Mood Inventory Scale (BMIS; Niedenthal and Dalle, 2001) and the Positive and Negative Affect Schedule (PANAS; Watson et al., 1988) consist of lists of emotional adjectives/emotions in which the participant must rate, on a numeric scale, to what extent he/she is feeling the corresponding emotion. Similarly, based on a dimensional emotion approach (e.g., Russell, 1994), the Pleasure Arousal Dominance scale (PAD; Mehrabian, 1996) requires participants to rate their level of pleasure, activation and dominance on a numerical scale. The Self-Assessment Manikin scale (SAM; Bradley and Lang, 1994) measures the same dimensions, but using non-numerical/non-verbal pictorial assessment scale, thus overcoming some difficulties often identified with the verbal scales (e.g., difficult to employ in childhood and for cross-cultural studies).

Despite all these instruments are well validated and currently employed in the clinical practice, they heavily rely on the person’s self-awareness, and thus may be biased by the instructions and the participant’s expectations.

### Emotion Regulation

ER refers to the processes meant to influence the intensity, duration and type of emotion experienced (Gross and Thompson, 2007). ER permits flexibility in emotional responding in accord with one’s momentary as well as one’s longer term goals in any given situation. For instance, ER can be useful to reduce the intensity and/or the duration of negative emotions, such as anger (Kashdan et al., 2015) or sadness (Millgram et al., 2015). ER comprises a set of different strategies, including for instance attentional deployment, in which the attention is shifted away or towards emotional aspects of a stimulus or situation (Manera et al., 2014), cognitive reappraisal, in which people change the way they think about a stimulus or situation in order to reduce negative feelings (e.g., Gross, 1998; Ochsner et al., 2002), and expressive suppression, in which people hide their emotions so that someone watching them would not know what they are feeling (Gross and Levenson, 1993; Lévesque et al., 2003). A variety of classification systems exist for emotion regulatory processes (e.g., Gross, 1998; Koole, 2009). For the purposes of the present article, a distinction particularly relevant is that described by Gyurak et al. (2011), referring to explicit (effortful) vs. implicit (automatic) ER processes. Following this classification, explicit emotion regulation (EER) processes require conscious effort for initiation, demand some level of monitoring during implementation, and are associated with some level of insight and awareness. EER are studied by asking participants to use a specific strategy, such as cognitive reappraisal, attentional deployment or expressive suppression to change the way they feel (Gross, 1998). EER abilities have investigated in an extensive corpus of literature (for reviews, see Gross, 2013), and through several tasks, ranging from self-report questionnaires (e.g., the ER Questionnaire, Gross and John, 2003), and the Kentucky Inventory of Mindfulness Skills (KIMS; Baer et al., 2004) to informant reports (e.g., the Weekly Coping Questionnaire, Khor et al., 2014), to naturalistic observation and behavior coding (see Weiss et al., 2014). Implicit emotion regulation (IER) processes are believed to be evoked automatically by the stimulus itself and run to completion without monitoring and can happen without insight and awareness. IER includes, among others, emotional conflict adaptation, habitual ER, emotion regulatory goals and values, and ER as a result of affect labeling (Gyurak et al., 2011), Surprisingly, IER strategies have received much less attention in the literature (Gyurak et al., 2011). This is unfortunate, as deficits in IER have important maladaptive consequences, as shown by the functional impairment of patients with generalized anxiety disorder (Etkin et al., 2010). Tasks employed to assess IEM include a few self-report questionnaires (e.g., Gross and John, 2003), and implicit tasks such as the emotional conflict task, employed to assess emotional conflict adaptation (Etkin et al., 2006). This is the emotional version of the classic Stroop paradigm (Stroop, 1935), and assesses IER through a progressive reduction in reactions times in trials in which there is a conflict between the photograph of an emotional faces (e.g., fearful) and the word (e.g., “happy”) written over it. The emotional conflict task has shown to be useful in clinical populations, such as patients with social anxiety disorders (Etkin et al., 2010). However, as the task relies on reaction times, it is not suitable to pathological populations with motor deficits, such as patients with Parkinson’s disease (PD), or to patients showing a slowdown in response due to psychomotor problems, such as affective deficits.

### Time Perception and the Internal Clock Theory

In Treisman (1963) elaborated his internal clock theory, which is the basis of all the current models of time estimation. He suggested to that our time measuring mechanisms can be described as an internal clock consisting of an arousal-sensitive pacemaker, which sends continuously and constantly pulses to a counter. The model also involves a store of “reference” durations, and a comparator mechanism. Subjective time estimations derive from the comparison of values in the counter and in the store. In this model, internal and external stimuli are supposed to modify the activity of the internal clock by modifying the activation of the physiological/arousal system, which affects the activity of the peacemaker. For instance, arousing emotional stimuli are supposed to speed up the pacemaker, which sends more pulses to the counter, thus resulting in a subjective time overestimation. Treisman’s internal clock model was remodeled by Gibbon’s scalar theory (Gibbon et al., 1977, 1984). Gibbon added to the basic architecture of Treisman’s model the idea of a switch, which can stop the pulse count and can be activated by attentional mechanisms, that is by cognitive factors. When the switch is activated, it prevents the pulses to enter into the accumulator, thus resulting in a time underestimation. Based on this model, we can thus assume that duration judgments reflect both a physiological, arousal-mediated component, and a cognitive, attention-mediated component. Several studies focused on divided attention have confirmed that, when participant’s attention is captured away from the time estimation task, it results in a time underestimation (Coull et al., 2004, 2011; Buhusi and Meck, 2005; Ulrich et al., 2006; Shi et al., 2013; Henry et al., 2015).

### Effect of Emotions and Attentional Processes on the Time Estimation

Consistent with the internal clock models, studies conducted so far demonstrated that emotional stimuli can result either in a time overestimation, either in a time underestimation depending on whether the emotional arousal component or the attentional cognitive component is prevailing (Lejeune, 1998; Elliott et al., 2000; Burle and Casini, 2001).

The arousal-elicited time overestimation has been studied since the 1960’s. Langer et al. (1961) were the first to show that inducing anxiety (by exposing participants to a dangerous situation) produced a temporal overestimation proportional to the intensity of the perceived danger. Based on the internal clock theory, it can be assumed that danger perception increases the pacemaker pulse rate, which thus generates a higher number of pulses that under normal conditions, resulting in a time overestimation. Watts and Sharrock (1984) confirmed these results studying patients suffering from arachnophobia. Similarly, it has been shown that stressful situations (such as the fear of electric shocks or of unpleasant social interactions) induce duration overestimates (for a review, see Hancock and Weaver, 2005). Also, several studies showed that stimuli depicting negative emotions are perceived as longer in duration compared to neutral stimuli (Wittmann et al., 2010; Grommet et al., 2011; Gil and Droit-Volet, 2011; for reviews, see Gil and Droit-Volet, 2012; Gros et al., 2015). Interestingly, the overestimation effect is stronger when emotions are presented in the context of a social interaction (Thayer and Schiff, 1975), thus suggesting that emotion-elicited time overestimates is not found only in laboratory settings, but can be reproduced in real-life situations.

Recent studies have also confirmed the involvement of the attentional system in emotion-related time estimates (Zakay, 1989; Zakay and Block, 1996; Liu et al., 2015). It has been repeatedly shown that the more our attention is captured by relevant aspects of a stimulus/situation, the more the time seems short (Macar et al., 1994). Angrilli et al. (1997), using emotional pictures, showed that the duration of highly arousing, unpleasant pictures was overestimated, whereas the duration of highly arousing pleasant pictures was underestimated. Inversely, when employing low arousing pictures, the duration of unpleasant pictures was underestimated, while the duration of pleasant pictures was overestimated. The authors interpreted this opposite direction of the valence effect as a function of arousal as evidence that two different mechanisms are involved: an attention-driven mechanism for low arousal, and an emotion-driven mechanism for high arousal. Also, Droit-Volet et al. (2010) showed that the duration of musical excepts was underestimated compared to the duration of neutral sounds, possibly due to the fact that music captures the attentional system more consistently. Zélanti and Droit-Volet (2012) have recently shown that children become more accurate in estimating the duration of sounds earlier compared to what occurs for images, a result which has been interpreted as evidence that visual stimuli engage the executive attentional system more consistently than auditory stimuli.

### The Priming Paradigm and the Clock’N Test

**Figure 1:**
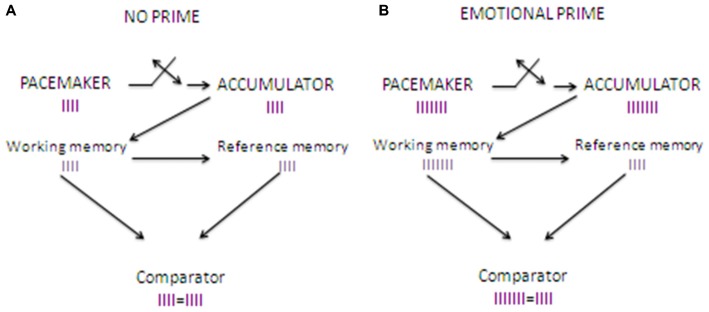
**Clock’N test: effects of the introduction of an odor emotional prime on time estimation**. **(A)** shows how time estimation works when no emotional prime is introduced. When the sound starts, pulses sent by pacemaker start entering the accumulator, and when the sound stops pulses stored in the accumulator (in the working memory) are compared to reference pulses stored in the long term memory. The comparator finds the same number of pulses in the working memory and the long term memory, and provides precise time estimation. **(B)** shows how time estimation works when an emotional stimulus such as an odor is presented as a priming stimulus before the time estimation task. When the sound starts the pacemaker, which is arousal-sensitive, starts to send pulses at a higher rate compared to baseline. When the sounds stops the pulses stored into the accumulator in the working memory are compared to reference pulses stored in the long term memory. As the number of pulses in the accumulator is higher compared to that found in the reference store, this results in a subjective time overestimation.

The priming paradigm is ideal to explore emotional processing in an implicit way, thus minimizing the role played by self-awareness and other high-level cognitive factors. The priming paradigm consists of presenting two stimuli sequentially, and observing the influence of the first (the prime) on the second (the target). Based on this paradigm, Gros et al. (2014, 2015) recently developed the The Clock’N Test, an emotional-priming time estimation task which was designed to study implicitly the effects of arousal-mediated and attention-mediated mechanisms on time estimation. In this test, participants have to estimate the duration of a neutral sound through a classical temporal bisection task (see below). Critically, the presentation of the sound is preceded by the presentation of emotional priming stimuli. In the first study, Gros et al. (2014) used olfactory stimuli as priming stimuli, which are well known to activate consistently the arousal system (Bensafi et al., 2002). Accordingly, they found an increase in skin conductance after the odor presentation, which was associated to a sound duration overestimation for all the presented stimuli, thus confirming that odors engaged the arousal-system consistently for the entire duration of the task (see Figure 1).

**Figure 2:**
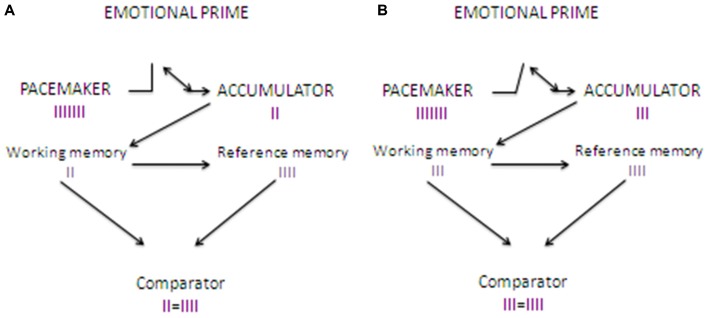
**Clock’N test: effects of the introduction of a emotional video prime on time estimation**. **(A)** shows how time estimation works when an emotional video stimulus is presented as a priming stimulus before the time estimation task. When the sound starts, the pacemaker, which is arousal-sensitive, starts to send pulses at a higher rate compared to baseline, as it happens for the odor priming stimuli. However, as video stimuli strongly engage attentional resources (devoted to the emotional aspects of the stimulus), the switch is activated, thus preventing pulses entering into the counter. When the sounds stops the pulses stored into the accumulator (in the working memory) are compared to reference pulses stored in the long term memory. As the number of pulses in the accumulator is lower compared to that found in the reference store, it results in a time underestimation. **(B)** shows how time estimation works after adaptation to the emotional video stimuli as a result of implicit emotion regulation (IER). Pulses are sent at the same rate as in **(A)**, but attention is progressively shifted away from the emotional aspects of the videos, thus allowing the switch to close later, and allowing more pulses entering into the counter. The result is that the comparator finds into the counter more pulses compared to **(A)**, but fewer compared to Figure 1A. The more the task goes on, the later the switch closes, thus allowing a progressive shift to time overestimation.

In their next study, Gros et al. (2015) compared the effect of odors and emotional videos priming stimuli. Confirming previous findings, odors resulted in a constant increase in skin conductance, accompanied by a constant time overestimation for the entire duration of the task. Interestingly, when videos were employed as priming stimuli, an initial increase in skin conductance was accompanied by sound duration underestimation. Critically, the level of physiological activation decreased progressively from the first to the last presented stimulus and was associated to a progressive shift from time underestimation to time overestimation. The results were interpreted as evidence that, at the beginning of the task, the emotional aspects of the videos captured consistently attentional resource, thus resulting in a time underestimation accompanied by high arousal. In terms of the internal clock theory, even if the pacemaker was accelerating and sending more pulses due to the emotional arousal, attentional mechanisms activated the internal switch, thus preventing the pulses to enter into the counter, and resulting in a time underestimation (Tse et al., 2004; Zakay, 2005). After the presentation of some stimuli, IER mechanisms reduced progressively the attention devoted to the emotional aspects of the videos and the participant’s arousal (which, however, never reached the zero). This allowed to re-open the switch, thus allowing the pulses to enter again into the counter, and to shift progressively towards the classical emotion-elicited time overestimation (see Figure 2).

In summary, even if evidence on discriminant and convergent test validity are still lacking, the task employing emotional videos as priming stimuli has been suggested to be useful to assess implicitly: (a) emotional reactivity, as indexed by the time underestimation observed in the first part of the task (and accompanied by high arousal), consequence of the attention devoted to the emotional aspects of the videos; and (b) IER abilities, as indexed by the progressive shift from time underestimation to time overestimation (accompanied by a progressive decrease in arousal), consequence of the habituation (reduction of the attention devoted) to the emotional aspects of the videos. Due to its implicit format, this task could be particularly suitable to study deficits in emotional reactivity and ER in clinical populations, such as patients with brain lesions affecting the arousal mechanisms (e.g., amygdala) or the regulation mechanisms (e.g., prefrontal regions). However, in order to be employed in the clinical practice, the test must be first validated on healthy participants, and normative data must be obtained for different categories of participants (e.g., males vs. females, elderly vs. young persons, etc.).

The purpose of the present article is to present normative data collected on a representative sample of healthy participants for the Clock’N test (video task). Study 1 was designed to replicate the results of Gros et al. (2015) on a bigger (*N* = 150) and more representative sample of participants, and to investigate the existence of effects of age, gender and laterality on the task. Study 2 was designed, first, to calculate meaningful scores to be employed in the clinical practice as proxy for emotional reactivity and IER ability; second, to provide psychometric indexes of the distribution of these scores in a representative population of healthy participants; and finally, to provide empirical cutoffs for these scores.

## Study 1

### Materials and Methods

#### Ethics Statement

Ethics approval was granted by the Ethics Committee Est I (France), and the study was labeled as non-interventional. A statement was made to the National Commission for Computing and Liberties by the Department of Clinical Research and Innovation by the Hospital of Dijon. Statement Number: 1758780v0, April 2014.

#### Participants

One hundred and fifty healthy adults participated in this study (68 M, 82 F; age range: 20–77 years; mean age: 46.5 years, SD: 17.0 years). They were all residents of the Burgundy region (France), and their mother tongue was French. One hundred and thirty three participants were right-handed, 12 participants were left-handed, and the remaining 5 participants were ambidextrous. Twenty four participants had less than 12 years of formal education (study certificate lower than the French BAC), 55 had 12 years of education (BAC certificate), 37 had 14 years of education (BAC+2 certificate), and the remaining 34 had 16 years of education or more (BAC+4 or more). Participants were not included if they had pathologies causing cognitive impairment (e.g., brain tumor, neurological disorder) and visual or auditory impairments. The Hamilton Depression Rating Scale (HDRS; Hamilton, 1980) was employed to evaluate the presence of depressive symptoms.

#### Tasks and Design

##### Video priming stimuli

The video stimuli were selected from the tool developed by Schaefer et al. (2010). We conducted pretests with more than 50 healthy participants, to select the films clips that were not traumatizing, but still able to generate a consistent electrodermal response. Seven films were selected: three films with positive valence and four with negative valence. In the original study, the valence (positive and negative) was assessed with a validated French translation (Gaudreau et al., 2006) of the PANAS (Watson et al., 1988). For each of the 20 emotion-related words, participants used a 5-point scale (1: “very slightly or not at all”, 5: “extremely”) to rate the extent to which they felt each emotion as they were watching the film clip. The selected video-stimuli were previously employed as priming stimuli by Gros et al. (2015), who confirmed that all the stimuli were arousing, and were able to produce a significant effect on target sound stimuli.

##### Target sounds employed for the time estimation task

The auditory stimuli employed for the time estimation task were generated with the software PRATT (LF-ARX model). This is an algorithm for the sound analysis/synthesis able to produce different voice components which has often been employed to explore the acoustic components associated to different emotions. Critically, the software allows generating pure sounds with the desired frequency and intensity. For the purposes of the present study, we selected a pure sound, which has the advantage of not being associated to any personal experience, as it does not exist in nature. The same sound was generated in seven different durations all shorter than 2 s (0.4, 0.6, 0.8, 1, 1.2, 1.4, 1.6 s). Indeed, we know that for sounds longer than 2 s we can use other strategies, such as counting, to increase the time estimation accuracy (Burle and Casini, 2001).

#### Experimental Design and Procedure

The study took place in a temperature controlled and well ventilated room at the Exploration Lab Nervous System of Dijon (France). The experiment consisted of two phases occurring sequentially. In the first phase (phase 1), we determined a time judgment baseline for each participant, as duration judgments are known to vary consistently across individuals, and are affected by multiple factors (e.g., time of the day, age, sex, attention; Lejeune, 1998; Bschor et al., 2004; Droit-Volet et al., 2007). In the second phase (phase 2), we explored whether the subjective time judgment varied with the presentation of olfactory or visual priming stimuli.

##### Phase 1: subjective time estimation

As temporal distortions can be caused by several factors, including individual differences (Bschor et al., 2004; Droit-Volet et al., 2007), before starting the task we create a subjective time estimation baseline for each participant, by asking the participant to judge the duration of the same sounds presented later in the priming task. Similarly we collected the basic electrodermal response of each participant because they can vary depending on many factors (such as age, sex, health status, etc.).

Participants were presented with a paper sheet with a 20 cm line with three anchor points indicating 0, 1 and 2 s. Before starting the time estimation task, participants were presented with two sounds of 1 and 2 s, to give them standard reference points. Participants were then presented with seven sounds of 400, 600, 800, 1000, 1200, 1400 and 1600 ms in the following order: 1400, 600, 1200, 400, 800, 1000 and 1600 ms. The task progression was the same for all participants. After each sound, participants were asked to bisect the line in front of them to indicate the estimated duration of the sound. They were given no feedback on their performance. These estimates were used as a baseline for the time estimation made in Phase 2.

##### Phase 2: priming task with video stimuli

**Figure 3:**
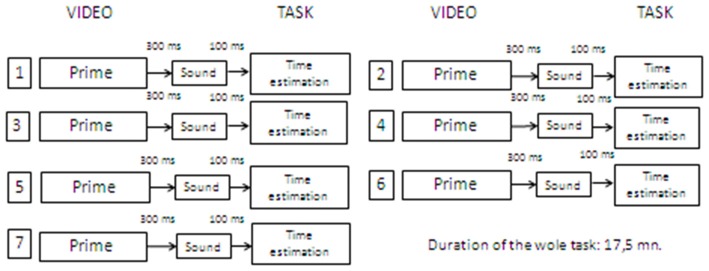
**Summary of the Clock’N test phase 2 procedure**.

Participants were asked to watch a short video-clip. After 300 ms from the video presentation (Hermans et al., 2001), participants were presented with a target sound and asked (after 100 ms) to estimate its duration with the same line bisection procedure used in Phase 1. The time interval between two different trials was one and a half minutes and equivalent across conditions. The procedure was repeated seven times, with seven different videos and the seven sounds employed in Phase 1 (presented in the same randomized order as in Phase 1; see Figure 3). Participants were not asked to provide ratings on the emotional priming stimuli because this would have focused their attention to the emotional aspects of the task. They were given no feedback on their performance at any time. The whole task took around 15–20 min to complete.

### Data Analysis

In order to obtain unbiased measures of time estimation, we calculated for each stimulus: (1) the *baseline time estimation*, i.e., the difference between time estimation provided in phase 1 and the real sound duration; (2) the *emotional priming time estimation*, i.e., the difference between time estimation provided in phase 2 and the real sound duration; and (3) the *time warp*, i.e., the difference between the time estimation provided in Phase 2 and the time estimation provided in Phase 1. The baseline time estimation, the emotional priming time estimation and the time warp were then submitted to a repeated-measure ANOVA with task progression (from the 1st to the 7th presented stimulus) as a within-subject variable and age (19–39 years, 40–60 years and 61–81 years), gender (male vs. female) and laterality (right-handed, left-handed and ambidextrous) as between-subject factors. The ANOVA linear contrast was also reported. Statistical analyses were performed using SPSS 20.0 (IBM).

### Results

#### Baseline Time Estimation (Phase I)

**Figure 4:**
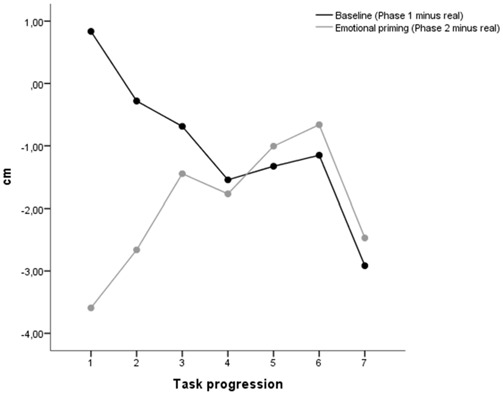
**Time estimates for Phase 1 and Phase 2 (difference compared to real sound duration)**.

A single sample *t*-test (test value = 0) conducted on the mean baseline time estimation (mean difference between time estimation provided in phase 1 and the real sound duration for the seven stimuli) revealed that, as a group, participants had a slight tendency to underestimate the sound durations (*M* = −1.01, *SD* = 2.46, *t*_(149)_ = −5.04, *p* < 0.001). A repeated measures ANOVA on the baseline time estimation with task progression as within-subjects factor revealed a significant effect of task progression (*F*_(6,894)_ = 41.89, *p* < 0.001), with the baseline time estimation decreasing linearly from the first to the last presented stimulus (linear contrast, *F*_(1,149)_ = 103.56, *p* < 0.001; see Figure 4).

##### Effect of age, gender and laterality

A repeated measures ANOVA on the baseline time estimation with task progression as within-subjects factor and age (19–39 years, 40–60 years and 61–81 years), gender (male vs. female) and laterality (right-handed, left-handed and ambidextrous) as between-subject factors confirmed the significant effect of task progression (*F*_(6,810)_ = 7.23, *p* < 0.001), with the baseline time estimation decreasing linearly from the first to the last presented stimulus (linear contrast, *F*_(1,135)_ = 6.13, *p* < 0.001). No main effect of age (*F*_(2,135)_ = 0.63, *p* = 0.534), Gender (*F*_(1, 135)_ = 0.25, *p* = 0.620) or laterality (*F*_(2,135)_ = 0.06, *p* = 0.947) was found. A significant interaction between task progression and age was found (*F*_(12,810)_ = 1.84, *p* = 0.039), with participants in the intermediate age group (40–60 years) showing a less linear decrease of the baseline time estimation compared to younger and older participants. No other interaction reached statistical significance (all *p*s > 0.193).

#### Emotional Priming Time Estimation (Phase 2)

A single sample *t*-test (test value = 0) conducted on the mean emotional priming time estimation (mean difference between time estimation provided in phase 2 and the real sound duration for the seven stimuli) revealed that, as a group, participants had a tendency to underestimate the sound durations (*M* = −1.94, *SD* = 2.77, *t*_(149)_ = −8.59, *p* < 0.001). A repeated measures ANOVA on the baseline time estimation with task progression as within-subjects factor revealed a significant effect of task progression (*F*_(6,894)_ = 28.26, *p* < 0.001), with the baseline time estimation increasing linearly from the first to the last presented stimulus (linear contrast, *F*_(1,149)_ = 34.24, *p* < 0.001; see Figure 4).

##### Effect of age, gender and laterality

A repeated measures ANOVA on the emotional priming time estimation with task progression as within-subjects factor and age (19–39 years, 40–60 years and 61–81 years), gender (male vs. female) and laterality (right-handed, left-handed and ambidextrous) as between-subject factors confirmed the significant effect of task progression (*F*_(6, 810)_ = 2.71, *p* = 0.013), with the baseline time estimation decreasing from the first to the last presented stimulus. No main effect of age (*F*_(2,135)_ = 1.02, *p* = 0.363), Gender (*F*_(1,135)_ = 0.07, *p* = 0.792) or laterality (*F*_(2,135)_ = 0.70, *p* = 0.496) was found. No two-way or three-way interaction reached statistical significance (all *p*s > 0.177).

#### Time Warp (Phase 2 Minus Phase 1)

**Figure 5:**
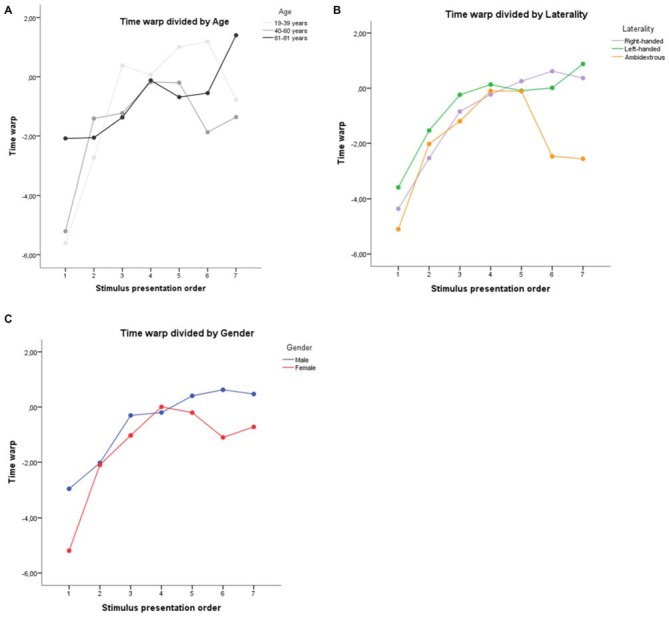
**Mean time warp for Study by (A) Age, (B) Laterality, and (C) Gender**.

The time warp (difference between time estimation provided in phase 2 and phase 1) divided by age, gender and laterality is reported in Figure 5. A single sample *t*-test (test value = 0) conducted on the mean time warp revealed that, as a group, participants had a slight tendency to underestimate the sound durations after emotional priming (*M* = −0.94, *SD* = 2.41, *t*_(149)_ = −5.40, *p* < 0.001). This is in line with the results obtained in our study report employing the same stimulus material. In line with the results of Gros et al. (2015), a repeated measures ANOVA on the time warp with task progression as within-subjects factor revealed a significant effect of task progression (*F*_(6, 894)_ = 59.79, *p* < 0.001), with the time warp increasing linearly from the first to the last presented stimulus (linear contrast, *F*_(1, 149)_ = 137.20, *p* < 0.001). In order to check whether the time warp change was driven by the duration of the target sounds, we recomputed the ANOVA ordering the stimuli based on the target sound duration (from the shorter, 400 ms, to the longer, 1600 ms). The linear contrast using the duration of the target sounds as a ordering variable was not statistically significant (*F*_(1, 24)_ = 0.12, *p* = 0.733), thus suggesting than the presentation order, and not the duration of the target sounds, was indeed the critical variable in generating the time warp shift.

##### Effect of age, gender and laterality

A repeated measures ANOVA on the time warp with task progression as within-subjects factor and age (19–39 years, 40–60 years and 61–81 years), gender (male vs. female) and laterality (right-handed, left-handed and ambidestrous) as between-subject factors confirmed the significant effect of task progression (*F*_(6,810)_ = 7.99, *p* < 0.001), with the time warp increasing linearly from the first to the last presented stimulus (linear contrast, *F*_(1,135)_ = 13.88, *p* < 0.001). No main effect of age (*F*_(2,135)_ = 0.28, *p* = 0.753; Figure 1A), gender (*F*_(1,135)_ = 0.02, *p* = 0.900; Figure 1B) or laterality (*F*_(2,135)_ = 0.63, *p* = 0.535; Figure 1C) was found. A significant interaction between task progression and age was found (*F*_(12,810)_ = 2.18, *p* = 0.011)^1^, thus confirming all the research suggesting a change in the emotional process during age. No other interaction reached statistical significance (all *p*s > 0.174).

###### Age

Contrary to what might have been expected from the literature (Sims et al., 2015; Scheibe et al., 2015), we did not find that older people had a faster shift to time overestimation compared to younger people.

###### Gender

Even if not statistically significant, it is noteworthy that the time underestimation at the beginning of the study was more pronounced in women compared to men. These results are consistent with previous reports that suggest a higher emotional reactivity among women (McManis et al., 2001).

###### Laterality

Data inspection revealed that the performance of the ambidextrous was clearly different from the right-handed and left-handed participants, especially in the last three stimuli, where they did not show any passage to a temporal overestimation (the absence of statistical significance can be explained by the low number of ambidextrous participants, *N* = 5). This finding is consistent with the literature that shows lower emotional reactivity in ambidextrous (Christman, 2014).

## Study 2

Based on the data collected in Study 1, the objective of Study 2 was to establish normative scores for the Clock’N test, to be employed in the clinical practice. As detailed in the introduction, the task employing emotional videos as priming stimuli involves (in our interpretation of the results) two different mechanisms: (1) an attention-related mechanism (i.e., the attention is captured by the emotional aspects of the video), which has a stronger effect in the first phase of the task, and results in a time underestimation associated to high arousal (as indexed by skin conductance, see Gros et al., 2015); and (2) an implicit emotion-regulation mechanism (IER, i.e., a decrease in the attention devoted to the emotional aspects of the videos), which results in an progressive shift from time underestimation to time overestimation over the second part of the task, with a correspondent progressive decrease in arousal.

The objective of the present study was to define two separate scores to be used as proxies of individual emotional reactivity and IER.

### Participants

In order to obtain a sample representative of the general population for age and gender, we referred to the INSEE data (results collected until the end of 2014), and we randomly selected from the sample collected in study 1 *N* = 137 participants, distributed as follows: 54 participants in the 19–39 years range (24 M, 30 F), 48 participants in the 40–60 years range (24 M and 24 F) and 35 participants in the 61–81 years range (16 M and 19 F). 20 participants had less than 12 years of formal education (study certificate lower than the French BAC), 49 had 12 years of education (BAC certificate), 36 had 14 years of education (BAC+2 certificate), and the remaining 32 had 16 years of education or more (BAC+4 or more). One hundred and twenty five participants were right-handed, and 12 participants were left-handed. Ambidextrous participants tested in Study 1 (*N* = 5) were excluded because they were too few, and because data inspection revealed a different performance in the last part of the experiment.

In order to check that the results found in Study 1 were replicated in the new sample, we repeated all the data analyses reported in the previous study on the new sample. No differences were found between the full sample used in Study 1 and the representative sample selected for Study 2.

### Definitions of the Scores

**Figure 6:**
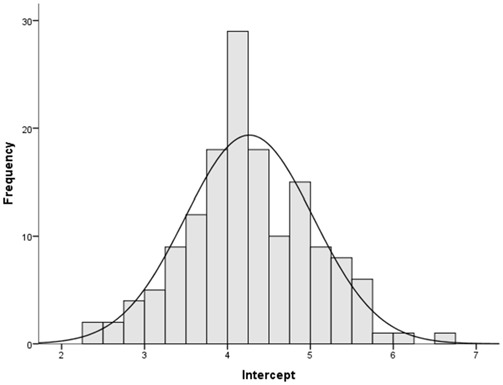
**Study 2: histogram showing when the shift from time underestimation to time overestimation did occur**.

In order to measure separately the two mechanisms of emotional reactivity and EIR, we first analyzed when participants, as a group, shifted from time underestimation to time overestimation. For each participant, we first fitted a regression line to the time warp data, putting the seven stimuli (ordered based on the original task progression) on the *y*-axis (ordinate), and the time warp on the *x*-axis (abscissa). We then calculated the line intercept using the Microsoft Excel “Intercept” function, to calculate the *y*-value (stimulus) for which the *x* (time warp) was equal to zero. Descriptive analysis revealed that, for more than 50% of participants, the passage from time underestimation to time overestimation took place around the 4th presented stimulus (*M* = 4.25, *SD* = 0.77; median = 4.23; see Figure 6). This result is consistent with the results of our previous study (Gros et al., 2015). Using the 4th stimulus as a cutoff, we then calculated the two following scores:

*Score 1:* mean time warp among the first three presented stimuli, which represents a measure of emotional reactivity; lower scores indicates higher emotional reactivity.
*Score 2:* difference between the mean time warp in the last three presented stimuli and score 1; higher scores indicates stronger EIR skills, given the presence of emotional reactivity (low score 1).

An alternative method to evaluate the EIR skills is to fit a regression line to the individual time warp data (putting the time warp on the *y*-axis and the seven stimuli ordered based on the original task progression on the *x*-axis) and calculate the line slope (for instance with the Microsoft Excel function “slope”). A higher slope indicates a faster shift from time underestimation to time overestimation, and thus stronger EIR skills. However, we decided to provide normative data only concerning Score 1 and Score 2 because they are easier to calculate, and thus can be used more extensively in the clinical practice.

### Normative Data

Descriptive analyses for score 1 and score 2 are reported in Table 1. Single sample *t*-tests (test value = 0) confirmed that Score 1 was significantly lower than zero (*t*_(136)_ = −10.64, *p* < 0.001) and that Score 2 was significantly higher than zero (*t*_(136)_ = 11.01, *p* < 0.001). Skewedness for both Score 1 and Score 2 was lower than one, thus indicating a symmetrical data distribution. Kurtosis values were above one for both Score 1 and Score 2, thus indicating a distribution slightly more peaked compared to a standard Gaussian distribution. Finally, in order to crosscheck whether age, gender and laterality had an effect on the two scores, we submitted Score 1 and Score 2 to separate between-subject ANOVAs with age (19–39 years, 40–60 years and 61–81 years), gender (Male vs. Female) and laterality (right-handed vs. left-handed) as between-subject factors. Confirming the results of Study 1, results revealed no significant effect on Score 1 (corrected model: *F*_(10,136)_ = 0.79, *p* = 0.636; Age: *F*_(2,126)_ = 0.82, *p* = 0.442; Gender: *F*_(1,126)_ = 0.61, *p* = 0.438; Laterality: *F*_(1,126)_ = 0.13, *p* = 0.718) or Score 2 (corrected model: *F*_(10,136)_ = 0.85, *p* = 0.580; Age: *F*_(2,126)_ = 0.46, *p* = 0.630; Gender: *F*_(1,126)_ = 0.40, *p* = 0.529; Laterality: *F*_(1,126)_ = 0.34, *p* = 0.561). For this reason, we did not provide separate normative data for different age, gender and laterality groups. Normative data on the time warp are presented in Table 2.

**Table 1:** **Descriptive analyses for score 1 and score 2**.

		Mean (*SD*)	Range	Skewness (*SE*)	Kurtosis (*SE*)
Score 1	cm	−2.58 (2.84)	−10.17–10.07	0.56 (0.21)	2.20 (0.41)
	s	−0.25 (0.28)	−2.03		
Score 2	cm	3.11 (3.30)	−6.83–14.00	0.35 (0.21)	1.18 (0.41)
	s	0.31 (0.33)	−2.08		

**Table 2:** **Clock’N test: normative data**.

		10%	25%	50%	75%	90%
Score 1	cm	−6.04	−4.48	2.63	0.63	0.67
	s	−0.6	−0.45	0.26	0.06	0.07
Score 2	cm	−0.71	1	2.77	5.12	6.95
	s	−0.07	0.1	0.28	0.51	0.69

As a rule of thumb, normative data for score 1 are usually below zero (in 85, 1% of the participants in our sample), and the normative data for score 2 are usually above 0 (in 86, 1% of the participants in our sample). It should be noted that score 2 is dependent on the results of score 1. Specifically, if score 1 is above 0 (no emotional reactivity), score 2 (EIR ability) should not be interpreted, as there was no emotion to regulate. More in general, comparisons of scores 2 among different individuals are meaningful only when their scores 1 are similar.

### Effect of Depressive Symptoms

In order to provide suggestions on the potential of the use of the Clock’N test in clinical populations, we performed an exploratory analysis to verify whether self-reported depressive symptoms (as indexed by the HDRS, 17-item version) had any effect on Score 1 and Score 2. ANOVA on score 1 with Depression (score 0–4, 5–9 and >9) as between-subject factor was not statistically significant (*F*_(2,130)_ = 0.07, *p* = 0.933), with the mean values for Score 1 being negative for the three Depression categories (score 1 ranging from −2.34 to −2.57). ANOVA on score 2 with Depression as between-subject factor was not statistically significant (*F*_(2,130)_ = 1.73, *p* = 0.181). However, the mean values for participants scoring higher than 9 in the HDRS (*N* = 2) were negative (*M* = −1.18), while they were positive, as expected, for participants scoring lower than 9 (group 0–4, *M* = 3.07, group 5–9, *M* = 3.38). The absence of statistical significance can be explained by the low number of participants in the high depressive symptoms group.

## Discussion

In the present study, we reported normative data for the Clock’N test (Gros et al., 2014, 2015), an instruments designed to assess implicitly emotional reactivity and IER abilities, and that has several potential application in the clinical practice. In the first study, we replicated the results of Gros et al. (2015) showing that, at the beginning of the task, participants underestimated sound durations, due to the attention devoted to the emotional aspects of the stimulus (Tse et al., 2004; Zakay, 2005).

In the terms of the internal clock theories, this effect of time underestimation can be interpreted as evidence that the attention devoted to the emotional videos closed the internal switch, thus potentially preventing the pulses to enter the counter. This result is consistent with the studies showing that stimuli capturing attentional resources result in a time underestimation (e.g., Angrilli et al., 1997; Droit-Volet et al., 2007; Noulhiane et al., 2007; Tipples, 2010; Liu et al., 2015). Specifically, it has already been shown that complex visual stimuli consistently capture attentional resources (Droit-Volet et al., 2010). This supports the interpretation that the videos employed in the Clock’N test captured attentional resources, especially at the beginning of the task. Around the 4th presented stimulus, participants showed a passage to time overestimation, which reflected the effect of IER abilities, allowing to progressively disengaging the attention from the emotional aspects of the stimuli, and thus resulting in the classical arousal-elicited time overestimation. The arousal-dependent time overestimation found at the end of the task is consistent with a number of previous studies. For instance, taking medications with an exciting effect induces a time overestimation (Meck, 1983; Wearden and Penton-Voak, 1995; Droit-Volet and Wearden, 2002). Studies employing audio stimuli (Droit-Volet et al., 2010; Cocenas-Silva et al., 2011) showed that musical excerpts with high arousal produced a time overstimation. Similar results have been found for stimuli presented in the visual modality (Nather et al., 2011; Gil and Droit-Volet, 2012; Fayolle and Droit-Volet, 2014), which systematically revealed that emotional pictures, which produced an increase in arousal, were systematically judged as longer compared to neutral pictures. In the terms of the internal clock theory, the effect of IER allowed to progressively open the switch, thus allowing the higher number of pulses induced by the increased arousal to enter again into the counter. Thus, the results of study 1 confirmed that the Clock’N test can be employed to assess both emotional reactivity and IER. Furthermore, in study 1 we showed that gender, laterality and age did not significantly affect the results of the Clock’N test. Despite these results were collected on a big sample of participants (*N* = 150), future research should remain open to the possibility of effects of age and laterality. Indeed, in the present study we did not test participants older than 77 years, and we know from literature that both emotional reactivity and ER undergoes a number of changes across the lifespan (e.g., Gosselin et al., 2002; Kalisch, 2012; Frankel et al., 2015; Scheibe et al., 2015; Sims et al., 2015). Similarly, we tested only five ambidextrous participants, and there is some evidence that emotional processing and ER may vary depending on the participant’s laterality (Christman, 2014; Rempala, 2014; Costanzo et al., 2015). Future studies should investigate whether including participants aged over 80 years and a more consistent number of ambidextrous participants can modify this pattern of results.

In study 2, we defined two separate scores to be used as proxies of individual emotional reactivity (Score 1) and IER (Score 2), and provided normative cutoffs for these two scores, based on the 10th and 90th score distribution percentiles. These cutoffs should allow clinicians to identify patients that have an emotional reactivity too low (Score 1 > 0.67) or to high (Score 1 < −6.04) compared to healthy participants, as well as to identify patients whose IER abilities are too low (Score 2 < −0.71) or too high (Score 2 > 6.95). Exploratory analysis confirmed that participants with reporting higher depressive symptoms, which are well known to affect IER abilities (Belden et al., 2015), had scores below the 10th percentile cutoff for Score 2, thus suggesting that these values may be useful to discriminate patients with specific impairments in IER. However, future studies including a bigger number of participants with high depressive symptoms are required to validate the provided cut-offs. As we described in the “Results” section, score 2 is dependent on the results of score 1. This means that, if score 1 is above 0 (no emotional reactivity), score 2 (EIR ability) should not be interpreted, as there was no emotion to regulate. This means that score 2 may not be adapted to test IER in patients with problems of emotional reactivity, such as patients with Alzheimer’s disease (AD; Lyketsos et al., 2002; Apostolova and Cummings, 2008), PD (Tessitore et al., 2002) or patients affected by stroke leading lesions of the basal ganglia (Onoda et al., 2011) and of the temporal lobe (Okada et al., 1997). More in general, comparisons of scores 2 among different individuals are meaningful only when their scores 1 are similar. In the next section, we will describe the categories of patients for which Score 1 and Score 2 may be more interesting to employ.

### Limitations and Future Research Directions

Despite our normative data were collected on a large and representative sample of participants, some limitation should be noted. First of all, we were unable to provide independent measures of test validity. For instance, we did not provide any measure for the test convergent validity (through correlation with existing tests supposed to measure the same abilities), nor for discriminant validity (through correlation with existing tests supposed to measure different abilities). This is due to the fact that, at present, there are no gold standards to assess experienced emotional reactivity and IER abilities. More evidence on the test validity needs to be collected to ascertain that the Clock’N test is indeed measuring emotional reactivity and IER abilities, as we suggest. This may be obtained by embedding the test in a battery of validated tasks assessing different aspects of emotional functioning. A second limitation is represented by the fact that we did not include patients with emotional deficits, and therefore we were unable to empirically verify the validity of the provided measures and of the cutoffs in discriminating these patients. We believe that it would be interesting to collect data on patients with diverse types of emotional deficits. For instance, it would be very interesting to test patients with brain lesions known to affect emotional reactivity (e.g., amygdala lesions) and IER (e.g., prefrontal lesions).

Concerning Score 1 (emotional reactivity), it would be interesting to test patients with brain tumors affecting the anterior cingulate cortex, who show deficits in the classical emotion recognition tasks (Baird et al., 2006). Using the Clock’N test in combination with emotion recognition tasks would allow to verify whether these two abilities are correlated, and to what extent loosing the ability to recognize other’s emotions relate to emotional reactivity. Patients with AD and PD would also represent interesting targets. It is well known that these patients are affected by apathy (Robert et al., 2006), and by a lack of reactivity to emotional stimuli (Lyketsos et al., 2002; Apostolova and Cummings, 2008). However, so far the deficits in emotional reactivity have been investigated only though self-reports (Bowers et al., 2006; Mograbi et al., 2012), which can lead to possible biases. Employing the Clock’N test in combination with the classical self-report measures would provide additional evidence for the hypothesis of an emotional reactivity deficit in patients with AD, and provide further evidence for the validity of the Clock’N test. Apathy and emotional blunting have also been reported in post stroke patients with basal ganglia (Onoda et al., 2011) and temporal lobe (Okada et al., 1997) lesions. The Clock’N test could allow verifying whether apathy in these patients is actually related with a lack of emotional reactivity, as it is commonly assumed. Finally, another category of interesting patients is represented by generalized anxiety disorders and bipolar disorders, which are characterized by emotional hyperactivity (Houenou et al., 2011), and thus would allow to verify if the upper limits of Score 1 are valid.

In terms of implicit emotional regulation (Score 2), it would be interesting to employ the Clock’N test in patients with bipolar disorders, characterized by dysfunctions of the prefrontal networks involved in ER (Phillips et al., 2003), and in patients with anxiety disorders (Etkin et al., 2010). In addition to psychiatric pathologies, ER deficits were also found in patients who suffered from stroke (Cooper et al., 2015) or in patients with tumors in the cerebellum (Hopyan et al., 2010).

Going beyond the classical self-report measures, we believe that the Clock’N test may provide clinicians with an additional, more objective measure to help detecting affective problems potentially underestimated, especially in those pathological populations for whom affective problems are not the key symptoms. Further validation and research will help to clarify the usefulness of this test in the clinical practice, and its potentials in suggesting new treatment options for deficits in emotional reactivity and ER.

## Author Contributions

Conceived and designed the experiments: AG, MG, YB. Performed the experiments: AG, AD, OR, SG, MLM. Analyzed the data: VM. Wrote the paper: AG, VM, YB, MG, AD, SG, OR, MLM. All authors listed, have made substantial, direct and intellectual contribution to the work, and approved it for publication.

## Conflict of Interest Statement

The authors declare that the research was conducted in the absence of any commercial or financial relationships that could be construed as a potential conflict of interest.

## Footnotes

^1^The results are the same if age (years) is used as a covariate in the Anova; no main effect of age is found *F*_(1,143)_ = 0.04, *p* = 0.836), while the interaction between age and task progression is statistically significant *F*_(1,143)_ = 5.88, *p* = 0.017).
